# Thermal Flow Sensors for Harsh Environments

**DOI:** 10.3390/s17092061

**Published:** 2017-09-08

**Authors:** Vivekananthan Balakrishnan, Hoang-Phuong Phan, Toan Dinh, Dzung Viet Dao, Nam-Trung Nguyen

**Affiliations:** 1 Queensland Micro- and Nanotechnology Centre, Griffith University, Brisbane 4111, QLD, Australia; vivekananthan.balakrishnan@griffithuni.edu.au (V.B.); hoangphuong.phan@griffithuni.edu.au (H.-P.P.); toan.dinh@griffithuni.edu.au (T.D.); 2 School of Engineering, Griffith University, Gold Coast 4222, QLD, Australia; d.dao@griffith.edu.au

**Keywords:** thermal flow, harsh environment, operational modes, transduction, materials, properties and packaging

## Abstract

Flow sensing in hostile environments is of increasing interest for applications in the automotive, aerospace, and chemical and resource industries. There are thermal and non-thermal approaches for high-temperature flow measurement. Compared to their non-thermal counterparts, thermal flow sensors have recently attracted a great deal of interest due to the ease of fabrication, lack of moving parts and higher sensitivity. In recent years, various thermal flow sensors have been developed to operate at temperatures above 500 °C. Microelectronic technologies such as silicon-on-insulator (SOI), and complementary metal-oxide semiconductor (CMOS) have been used to make thermal flow sensors. Thermal sensors with various heating and sensing materials such as metals, semiconductors, polymers and ceramics can be selected according to the targeted working temperature. The performance of these thermal flow sensors is evaluated based on parameters such as thermal response time, flow sensitivity. The data from thermal flow sensors reviewed in this paper indicate that the sensing principle is suitable for the operation under harsh environments. Finally, the paper discusses the packaging of the sensor, which is the most important aspect of any high-temperature sensing application. Other than the conventional wire-bonding, various novel packaging techniques have been developed for high-temperature application.

## 1. Introduction

**Figure 1:**
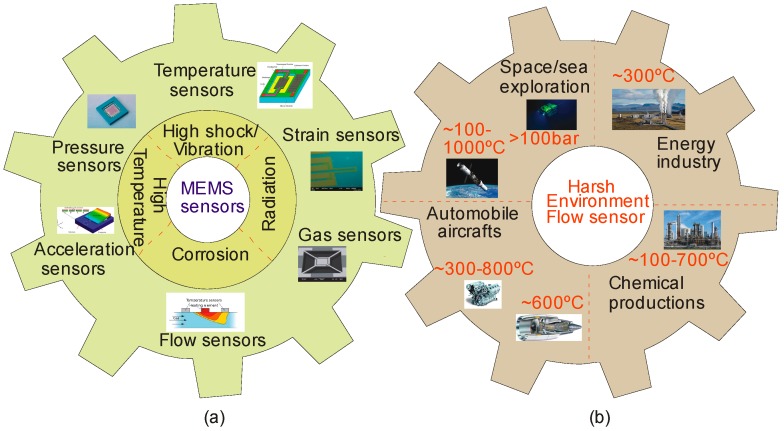
(**a**) Various MEMS sensors and harsh environments; (**b**) Applications of MEMS flow sensors and their corresponding conditions.

Micro-Electro-Mechanical Systems (MEMS) sensors such as pressure, temperature, strain, acceleration and flow are essential for many harsh environment applications. Harsh environments include but are not limited to high pressure, high temperature, corrosive and erosive nature that can hinder the operation of the device [1]. Figure 1a represents various MEMS sensors and the associated harsh environment conditions. These conditions are typically found in combustion optimization and emission control, oil industries, chemical process control, the propulsion systems of spacecraft and deep-sea devices [2,3]. In recent years, a number of flow sensors have been fabricated and characterized for the measurement of pressure [4,5,6], temperature [7,8], gas species [9,10,11,12,13], strain [14,15,16,17] and acceleration [18,19] in these environments. In addition, there has been growing demand for developing high-temperature miniaturized flow sensors with fast response times and low cost through mass fabrication. Figure 1b depicts the various applications of MEMS flow sensors and their operating ranges. For instance, automobile aircraft and propulsion system conditions are usually monitored by high temperature thermal sensors, which contribute to optimizing the operation conditions from 300 to 800 °C. In chemical production and oil and gas industries, where the operating temperature may range approximately from 100 to 700 °C, in-situ real-time monitoring of process parameters such as temperature, pressure and flow is indispensable as it contributes to the efficiency and process quality assurance [20]. Delivering science instruments through sulfuric acid clouds to land on the surface of space based environment such as Venus, where the pressure is more than 100 bar will inevitably require the use of MEMS flow sensors. Also, energy and automotive combustion systems require precise control of temperature, pressure and air-fuel ratio to obtain optimal efficiency and reliability [21].

Thermal flow sensors more often find applications in harsh environments than non-thermal flow sensors because of their fast response and the lack of moving parts. The advances in Micro-Electro-Mechanical Systems (MEMS) technology allows for the fabrication of miniaturized thermal flow sensors with extraordinary performance in terms of sensitivity, response time, cost-effectiveness and power consumption [22]. Among the various semiconductor sensors, silicon-based sensors play a significant role in many applications and numerous silicon flow sensors have been developed [23]. Nguyen et al. designed a silicon-based thermal mass flow sensor for different fluids and variable ranges emphasizing the need of mass flow measurement in various applications [24]. However silicon-based sensors cannot operate at high temperatures above 500 °C for a long period of time due to the degradation of silicon at high temperatures. In addition, the requirement of bulk housing in thermal sensor typically raises the cost of the sensors.

To resolve the limitations of silicon, alternative materials have been utilized in harsh environment sensors. For instance, silicon carbide (SiC) has gained much attention for sensing applications in harsh environments. Silicon carbide has high hardness, good thermal conductivity and is also chemically inert. Additionally, SiC has readily available large-scale commercial wafers, advanced MEMS processing technologies which allow rapid growth in the MEMS market [25]. Furthermore, gallium arsenide (GaAs), gallium nitride (GaN) and aluminium nitride (AlN) are also considered as MEMS materials for certain high-temperature applications. For instance, the typical operating temperature of gallium nitride is less than 700 °C, above which the material will degrade [26]. 

In the past two decades, research groups around the world have developed a number of thermal flow sensors for various applications. These works have attracted further interest from the research community in developing thermal flow sensors for niche applications that possibly impact the future MEMS market. However, there has been to date no comprehensive article reviewing the progress in the development of harsh environment thermal flow sensors.

## 2. Thermal Flow Sensors and Their Operation

### 2.1. Theory

The working principle of a thermal flow sensor is based on the heat exchange between the sensing element and the surroundings through forced convection. Without fluid flow, the heat exchange of a heater to the environment relies mainly on natural convection and conduction. The resulting temperature distribution around the heater is symmetrical. In contrast, a fluid flow removes the heat from the sensing element and the symmetry of the temperature field around the heated element is broken, resulting in an asymmetrical temperature distribution along the flow direction. The fluid velocity and the flow direction can be measured by the amount of heat removed from the heater and the temperature distribution around the heater.

**Figure 2:**
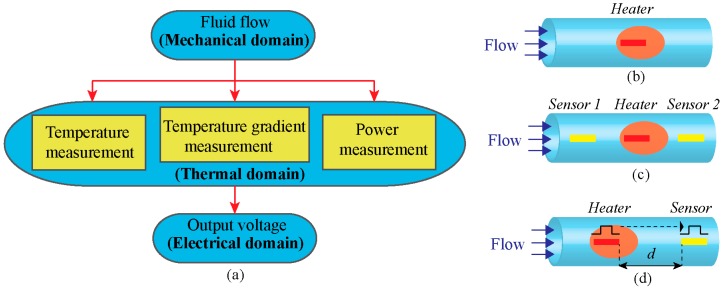
Thermal flow sensor, (**a**) Signal transduction path; (**b**) Hot-wire and hot-film configuration; (**C**) Calorimetric configuration; (**d**) Time of flight configuration.

The signal transfer process of a thermal flow sensor occurs in three domains: mechanical, thermal and electrical as depicted in Figure 2a [27]. The change in fluid flow (mechanical domain) leads to a temperature variation of the sensor (thermal domain) which can be converted to a voltage signal (electrical domain) [28]. According to the underlying physical principle, the thermal flow sensors operate in three configurations: (i) hot-wire or hot-film (Figure 2b), (ii) calorimetric or thermo-transfer (Figure 2c) and (iii) time of flight or thermal tracing (Figure 2d).

#### 2.1.1. Hot-Wire and Hot-Film Flow Sensors

Thermal flow sensors that sense the cooling effect of fluid flow through convective heat transfer on a heater are called hot-wire or hot-film sensors [29,30]. This configuration allows measuring a wide range of flow velocities based on the heat loss described as follows [31,32]:

(1) $$\frac{P}{\Delta T}=A+B\sqrt{U}$$

where *A* and *B* are constants that depend on the material respectively. U , P and ∆T represent the fluid velocity, power consumption and heater temperature relative to ambient, respectively [33]. 

**Figure 3:**
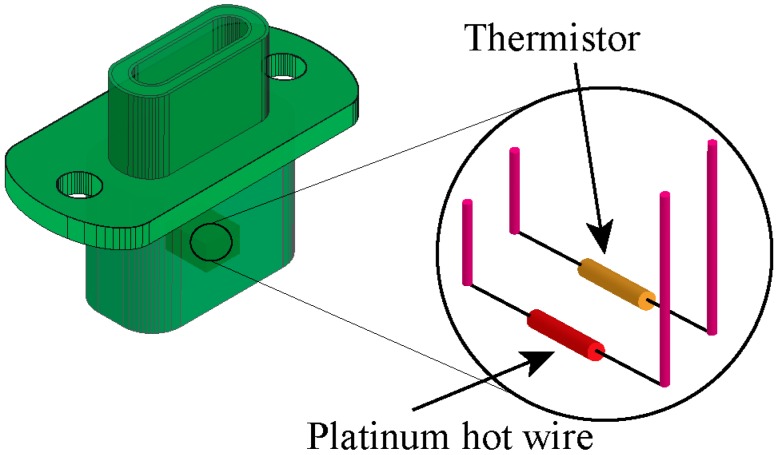
Mass air flow sensor for Toyota vehicles.

Hot-wire and hot-film sensors operate either in the constant power (CP) mode or in the constant temperature difference (CT) mode. CP mode detects the temperature change of the heater at a constant heating power. In CT mode, a servo-amplifier circuitry is used to establish a constant temperature between the probe and the ambient. Details about the use of these modes can be found in the literature [34,35,36,37,38,39]. As an early development, Toyota has developed a hot-wire flow sensor device, operated in CT mode in the fuel injection system. The amount of current supplied to hot-wire is proportional to the mass flow rate and the intake air temperature is monitored by a thermistor depicted in Figure 3. In this environment, hot-wire sensors are susceptible to dirt, smoke and oil particles present along with flow and subsequently the device leads to failure if contaminated.

Moreover, the use of hot-wire resistors at high temperatures for long periods needs to consider two important things: (i) the suppression of resistance change over the time when exposed to an impurity or dirt and (ii) the dependence of the Temperature Coefficient of Resistance (TCR) on impurity concentration. For instance, impurity diffusion on a resistor would affect its stability and cause drift under harsh conditions. This drift will adversely affect the sensitivity of a hot-wire flow sensor as TCR and sensitivity are closely related [40].

#### 2.1.2. Calorimetric Configuration

Thermal flow sensors that measure the asymmetry of the temperature profile around the heater are called calorimetric flow sensors [41]. A calorimetric configuration has a central heater (typically a thin film resistor) and two temperature sensors positioned upstream and downstream of the heater as shown in Figure 2b [42]. The output of a calorimetric thermal flow sensor depends on the specific heat capacity of the fluid. Under room conditions, the heat capacity is independent of temperature. However, it increases by 10% at 500 °C. Therefore, for harsh environment applications, the change of heat capacity has to be taken into account [43,44].

Examples of calorimetric flow sensors can be found in aerospace and automobile applications, where these sensors are capable of operating at elevated temperatures. Further features of these sensors are low cost and lower power consumption. For instance, Furjes et al. optimized the device geometry for measuring explosive gas mixtures, without the risk of exceeding the explosion limit. This gas flow sensor reduced the power consumption and can work at a maximum temperature of 500 °C [45].

**Figure 4:**
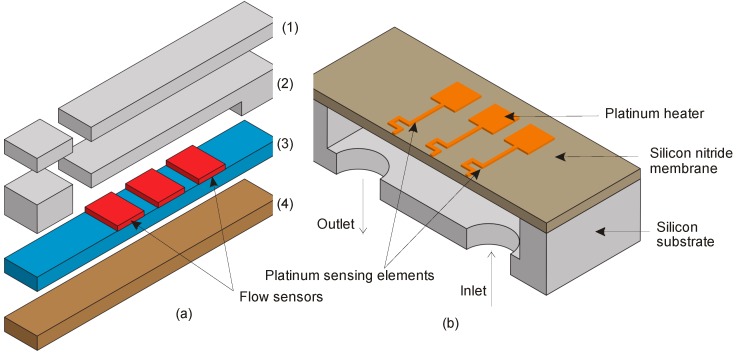
(**a**) Simplified stack segmented of thermal flow sensor developed by Lekholm et al. (1) Top layer for electrical and gas connections; (2) Second layer with gas channels, nozzles and vias for electrical connections; (3) Third layer with two propellant flow sensors, each containing a thruster and a central heater; (4) Stability layer during fabrication; (**b**) Calorimetric flow sensor reported by Palmer et al. where section along the channel of a device contains three platinum meanders placed on top of the silicon nitride membrane. Vias through the bottom silicon wafer serve as inlet and outlet to the gas channel etched in the top silicon wafer.

Lekholm et al. reported another interesting flow sensor, which employs a platinum heater and yttria stabilized zirconia temperature sensors (Figure 4a). The flow sensor, developed for thruster and aircraft applications, demonstrated an excellent sensitivity at 1000 °C [46]. Figure 4b shows an alternative kind of design, which separates sensing and heating elements by a silicon nitride membrane, which could measure corrosive gases with a flow range up to 300 standard cubic centimetres per minute (sccm) [47].

#### 2.1.3. Time of Flight Flow Sensor

Time-of-flight (TOF) sensors measure the time elapsed between the injection and the detection of a heat pulse [48]. This configuration consists of an upstream heater, which generates a heat pulse and transfers it to the surrounding fluid flow and at least one temperature sensor, which acts as a downstream sensor as shown in Figure 2d. The underlying fundamentals of TOF sensing have been intensively covered in [49]. 

This type of thermal flow sensor is useful for industrial applications that require measuring a process parameter over an extended period of time. Among the harsh environment applications, nuclear power plants have a great demand for measuring the coolant flow rate over a given time period. Moazenni et al. proposed a theoretical analysis to measure the flow rate using cross-correlation technique [50]. Moreover, thermocouples with grounded stainless steel shielding employed in this method has been so far the most robust and accurate solution to measure the thermal signal time. However, the use of stainless steel in micro-reactor/plant systems will be less effective towards corrosion resistance and the possible option could be the use of insulators such as glass in wetted areas and by placing sensing elements in non-wetted areas. Furthermore, in process/chemical industries, the time taken by the sensor to respond to a signal is a crucial parameter and it should be as short as possible. A few works on thermal time of flight sensors to achieve a response time on the order of milliseconds and a wide range flow measurement were reported in [47,51].

## 3. Transduction Mechanisms of Thermal Flow Sensors

According to the transduction methods, thermal flow sensors can be categorized into different types. Thermoelectric sensors employ thermopiles to detect the temperature difference between the ends of two different conducting materials. Thermoresistive sensors measure the temperature- dependent resistance. Thermoelectronic sensors operate based on the temperature dependence of diodes and transistors. The performance of various thermal flow sensing principles remains the same, with the differences being attributed to corresponding temperature sensors. Therefore, the knowledge of thermal sensors beforehand in the context of elevated temperatures is paramount.

### 3.1. Thermoelectric Flow Sensing

**Figure 5:**
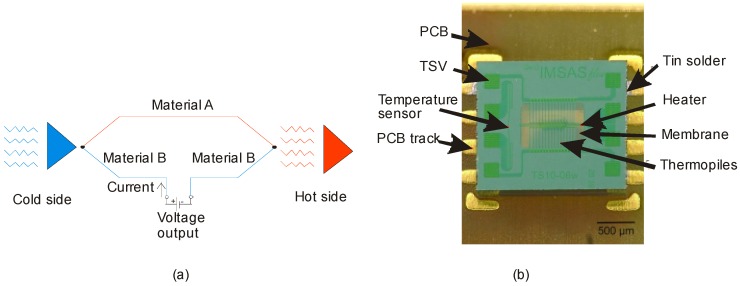
(**a**) Schematic illustration of a thermocouple; (**b**) Thermocouple based flow sensor reported by Sosna et al. [52] (Reprinted with permission from IEEE).

When two different metals are joined together on both ends to form a closed circuit, and one of the junctions is at a higher temperature than the other, a voltage is generated as shown in Figure 5. The phenomenon is called the thermoelectric effect. The temperature sensing elements are thermopiles, which is a number of thermocouples connected in series. Flow characterization using thermocouples is challenging as many process parameters have be considered. Furthermore, the thermocouples must be carefully protected from the harsh atmospheres due to the potential for corrosive attack. Moreover, fluid flow is difficult to measure as it changes with temperature and pressure constantly. Therefore, characterization of a flow sensor at room temperature can be an initial task to get an insight on the parameters such as Seebeck coefficient, electrical resistivity and thermal conductivity.

For a thermoelectric flow sensor, the thermopile efficiency greatly influences the sensitivity. The Figure of Merit, which depends on the above parameters quantifies the efficiency of converting thermal energy into electrical energy. Lei et al. reported the thermoelectric figure of merit for a n-SiC material to $4.6\times 10^{-6}$ which is superior to typical metals used in harsh environments such as platinum and gold, respectively [44]. In addition, this work has drawn three important conclusions on the performance of flow sensor at elevated temperatures: (i) the upstream thermopile voltage decreases with increasing operating temperature. Increasing temperature decreases the heater resistance and so as the heating power, (ii) the Seebeck coefficient increases with increasing temperature and (iii) the specific heat capacity changes at high temperatures around 300 °C. However, the primary drawback of this work is the sensor operating mode. In the constant-current mode, thermopiles produce different initial voltages at different temperatures, which can be problematic at high temperatures.

In contrast, constant-temperature-difference (CTD) mode can prevent this problem. Sosna et al. reported a miniaturized thermal flow sensor based on this mode. Figure 5b shows a flow sensor with a tungsten-titanium heater placed between two polysilicon/tungsten-titanium thermopiles respectively. Through Silicon Vias (TSV) enable the electrical connection between the sensor and the silicon chip on the bottom side. The sensor is bonded to the printed circuit board (PCB) using tin-solder. The sensor showed a good sensitivity under air flow suggesting the potential use for industrial applications.

### 3.2. Thermoresistive Flow Sensing

The thermoresistive effect is the change in electric resistance with temperature. Sensors that possess this behavior are called thermoresistive sensors or thermistors. Thermoresistive materials such as single crystal silicon, polycrystalline silicon or metals and alloys have been commonly employed for flow sensing thanks to their high thermoresistive sensitivity. Thermistors are categorized according to the sign of their temperature coefficient of resistance (TCR): negative temperature coefficient (NTC) and positive temperature coefficient (PTC) sensors. A thermistor can be used directly as hot-wire or hot-film sensors. Depending on the materials preferred, the R-T relationship can be linear/non-linear. The non-linear relationship between the resistance and temperature of a semiconductor thermistor is given by [53]:

(2) $$R=A\exp\left[B\left(\frac{1}{T}-\frac{1}{T_{o}}\right)\right]$$

where *A* is a constant and *B* is thermal index, which defines sensitivity of thermoresistive effect in thermistors. *T* and $T_{O}$ are the desired temperatures and room temperatures, respectively. The commonly employed thermistors offer many advantages such as high sensitivity and signal-to-noise ratio (SNR), and higher capability of temperature and simple probe assembly. The stability of a thermistor is measured by drift and it is quantified as a change in resistance that occurs at a given exposure temperature and duration. In extremely harsh environments, thermistors undergo drift as the limit of temperature exposure and length of the time increases [54]. More importantly, the operation of thermoresistive flow sensors at high temperatures is a challenging task with many factors to be considered such as: (i) the stability of the sensor; (ii) quick response time; (iii) low power consumption; (iv) superior sensitivity; (v) low cost; and (vi) small size. These factors are primarily determined by the choice of the material as: (i) it reduces the drift and provides less sensitivity to environmental effects; (ii) excellent mechanical properties such as high elastic modulus and yield strength providing a robust resistive bridge during the compressive fluid flow; and (iii) it avoids the breakage of bridges due to oxidation at high temperatures.

### 3.3. Thermoelectronic Flow Sensing

Thermoelectronic sensors employ bi-polar junction transistors (BJT), field-effect transistors (FET), metal-oxide semiconductor field effect transistor (MOSFET) and junction diodes as the sensing elements. These sensing elements are sensitive to temperature variations. In diodes, more hole-electron pairs are generated with increasing temperature (or thermal energy), leading to the excitation of diode conduction and an increase in the measured current. In a p-n junction diode, the current *I* at a given bias voltage *V* can be expressed as:

(3) $$I=I_{o}\left(e^{\frac{V}{\eta V_{T}}}-1\right)$$

where *I* is the current, $I_{o}$ is the reverse saturation current, *V* is the voltage across the diode, η is the ideality factor and $V_{T}$ is the voltage equivalent of temperature. The saturation current could be expressed by the following equation:

(4) $$I_{0}=C\sqrt{Tn_{i}^{2}}=CT^{\eta }e^{\frac{-qV_{G}}{V_{T}}}$$

where *C* is a constant that includes the density of states, effective masses of electrons and holes, carrier mobility, doping density, junction time and recombination lifetime. *η* is a process-dependent parameter (Si~3.5), $V_{G}$ is the extrapolated bandgap voltage at 0°K and $\eta_{i}$ is the intrinsic carrier concentration in the semiconducting material. The temperature of the voltage drop over a forward biased-emitter junction can be given by [55]:

(5) $$V_{BE}=V_{G}+\left[\frac{KT}{e}\ln\left(\frac{I_{C}}{I_{R}}\right)\right]$$

where $V_{BE}$ is the base-emitter voltage, $V_{G}$ is the band-gap of the material, *K* is the Boltzmann constant, *e* is the charge of an electron, $I_{C}$ is the collector current and $I_{R}$ is the reference current.

The presence of temperature sensors in a multi-sensor structure needs to fulfill two important requirements to function at high temperatures: (i) surrounding temperature monitoring and compensation employing a sensor off the membrane and (ii) heating element and temperature monitoring employing the sensor on the membrane. The challenge here is, given that the heating elements operate hundreds of degree Celsius, the temperature sensors need to withstand in excess of 300 °C. To meet this challenge, diode as a temperature sensor is a more suitable device than others as (i) it is extremely small in size; (ii) conduction losses to the substrate are minimized as they do not provide thermal bridge between the hot and cold zones of the chip; (iii) they offer wide range of temperature measurement; (iv) sensitivity enhancement can be easily obtained under high flow velocities by realizing in array form [56] and (v) strong temperature dependence on their forward bias voltage drop (4.2–888 K) [57]. To the contrary, transistors have also been used in anemometric configurations, often because of they are accurate absolute temperature sensors. This mode of operation usually has two transistors with the hot transistor measuring the temperature of the heated resistor surface modulated by the fluid flow and the cold transistor measuring the ambient temperature. The impact of high temperatures on the physical properties of various thermal sensors are summarized in Table 1.

## 4. State of the Art Materials and Properties for Harsh Environments

With the sensing effects and the transduction principles discussed in previous sections, choice of the materials is the next step towards designing thermal flow sensors. Materials are selected for heating, sensing and insulating elements. This section discusses the heating and sensing materials such as metals and alloys, semiconductors, ceramics and polymers, which are then followed by insulating substrate materials.

### 4.1. Heating and Sensing Materials

#### 4.1.1. Metals and Alloys

Metals can be deposited as a thin film on a chip to make a complete resistive sensing or heating element. These elements are made of many types of metals such as platinum, titanium, aluminum, and chromium. The choice of thin-film heating elements depends on various physical, chemical and electrical parameters. Platinum micro-heaters have been commonly employed as the resistive sensing and heating element because of their chemical stability at high temperatures and the simple micromachining process. However, platinum cannot withstand more than 850 °C. Usually, platinum is accompanied by a thin adhesive layer of titanium. The diffusion rate of this layer is high above 550 °C and decays the platinum layer [62].

Among the thermoresistive materials, nickel has also been preferred as it is much cheaper than platinum. And at the same time, the TCR of nickel is twice as high as that of platinum. However, the stable operating temperature of nickel is lower than that of platinum [63]. Nickel is suitable for good sensitivity and low cost applications at temperatures ranging from −100 °C to 200 °C. Chromium has also been chosen as heater material for some applications owing to its low temperature coefficient of resistance. On the contrary, gold has been a choice of interconnecting track metal due to smaller parasitic power losses and high stability [64].

Among thermoelectric materials, high-temperature stable alloys such as tungsten/titanium (WTi) along with polysilicon have been widely used. These together provide a high Seebeck coefficient value of $166\;\mu {\mathrm{VK}}^{-1}$ for each thermocouple thereby, making them as one of the excellent materials for temperature sensing [65]. Furthermore, the WTi alloys have also been used as a heater material since it possesses an almost constant temperature coefficient of resistance. Table 2 summarizes the key advantages and limitations of the high temperature metal alloys on the context of electrical, mechanical and physical characteristics(adapted and updated from [38,66,67].

#### 4.1.2. Semiconductors

Various semiconducting materials have been used for making a thermal flow sensor. The commonly used substrate material is silicon, which is superior to other materials in terms of properties and low cost due to the established microelectronics manufacturing processes. For instance, the sensitivity and reproducibility of silicon is higher than metals as reported by Billat et al. [68]. However, the application of silicon-based devices in harsh environments is limited due to its properties [69]. Amorphous germanium has been used as a highly sensitive thermoresistive material. The temperature resolution is on the order of 100 μK. The temperature coefficient of resistance is approximately −1.8%/K, which is five times higher than platinum making it as a suitable candidate for thermistors [70]. At high temperature, germanium is chemically less stable than platinum [71].

In some applications, the interconnection tracks were made of semiconducting materials such as polysilicon. Recently, Brushi et al. reported that the static thermopile voltage $V_{k}(0)$ is strongly affected by thermopile-heater spacing for various heater interconnection materials [22]. Polysilicon has been widely used as heater and temperature sensing material for the detection of hazardous gases in automotive exhaust control. The primary advantage of using polysilicon compared to platinum is that it neither catalyzes any reactions nor requires an adhesive layer [72]. Polysilicon could reliably operate up to 927 °C, suggesting that it could also be a potential candidate.

Silicon carbide (SiC) is a material known for its excellent electrical, mechanical and chemical properties [73]. Particularly, the high stability, toughness and hardness of SiC makes it a promising candidate for high temperature and high-frequency applications [74]. Furthermore, SiC has several advantages over other wide-bandgap semiconductors because of the available processing techniques and the ability to grow a thermal oxide for use as masks. Among over 100 polytypes of SiC, 3C, 6H and 4H SiC are commonly used in electronic devices due to their overall superior properties [75].

**Figure 6:**
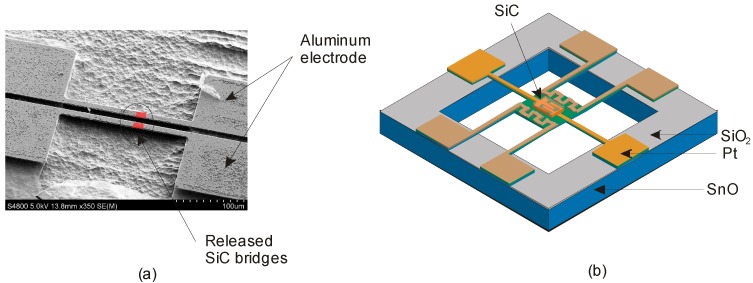
(**a**) Suspended silicon carbide resistor; (**b**) Design of high temperature micro-heater chip based on suspended β−SiC membrane.

Recently, SiC has been proven to shave excellent characteristics for high temperature thermal flow sensing thanks to its large bandgap and chemical inertness. For example, Phan et al. investigated the thermoresistive properties of suspended silicon carbide heater bridges as illustrated in Figure 6a. The observed high TCR indicated the possibility of using SiC as a thermal sensor [76]. Furthermore, Spannhake et al. made a major contribution by extending the temperature limitations of micro-heater devices, which silicon nitride or silicon oxide thin films as thermally insulating membranes, Figure 6b. In this work, the ability to deposit β-SiC directly onto the Si wafers using methylsilane was successfully presented, enabling long-term stable operation of micro-heating devices up to 1000 °C [77].

Another new material that has started to open up new applications in the field of high-temperature microdevices is antimony doped tin oxide. Spannhake et al. investigated the stability of microheaters around 950 °C. The lifetime of a heater made of antimony doped tin oxide could be of the order of 10 years and it does not suffer from oxidation in air. This feature avoids the problems during the packaging process of a heater element. In contrast, metallic heaters such as platinum suffer this problem. Moreover, metallic structures failed the stability test and this work clearly proved the superior performance of semiconductor heaters compared to the metallic counterparts. Table 3 summarizes the properties of various semiconductor materials at harsh environments.

Additionally, semiconductors such as alloys of bismuth telluride and bismuth selenide possess the largest figure of merit for p and n type materials up to 250 °C. The figure of merit and the maximum temperature at which it occurs can be modified through tuning of carrier concentration. Conventional thermoelectric materials that are operated in the range of 200 °C–600 °C are lead telluride and skutterudites, respectively [78,79]. Moreover, semiconductors such as silicon germanium with operating temperatures higher than 600 °C are also a good choice of thermal sensing elements.

#### 4.1.3. Polymers

The two important environments where polymers are subjected to extreme stresses are aerospace and geothermal applications. A strong coating, adhesives and sealants are required in these two environments for the flow sensors to function without any damage. For instance, an aerospace application typically requires polymers with resistance to extreme oxidation effects and better strength-weight ratio. Similarly, a geothermal application would impose severe effects such as abrasion and hydrolysis [80]. Nonetheless, only a few polymer based thermal flow sensors have been reported in recent years as the development is still in its early stages. Considering their excellent mechanical properties, more developments are expected in the forth-coming years. 

Buchner et al. developed a thermal flow sensor using two different polymer materials namely Delo Kationbond 4653 and Vitralit 1600 LV. The polymer is filled in the backside cavity of the sensor to stabilize the silicon nitride membrane so it can withstand the high pressure of 650 kPa. Moreover, the designed sensor shows high sensitivity and good dynamic behavior even under rough pressurized environments [83].

**Figure 7:**
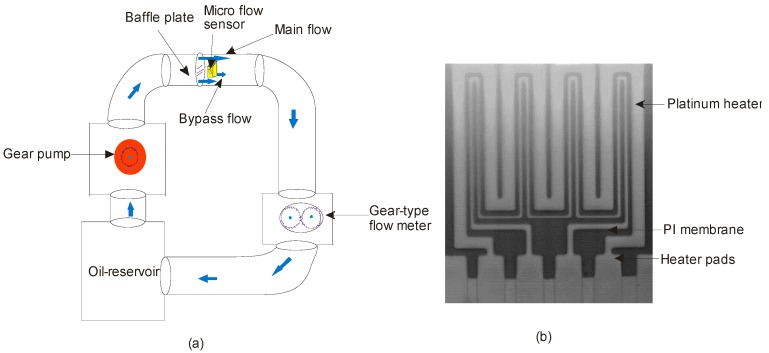
(**a**) Polymer-based microflow sensor for hydraulic systems; (**b**) Platinum heater on a Polyimide (PI) membrane reported by Aslam et al. [82] (reprinted with the permission from *Sens. Actuat. Chem. B*).

Another polymer called polyimide (PI) has been widely used as a membrane material to study the dynamic characteristics of flow sensors under high pressure. Excellent chemical resistance and thermal stability of PI make it popular as a membrane material for most of MEMS applications. For instance, Ahrens and Festa proposed a system that splits the micro flow sensor embedded on a PI membrane. The anemometric based micro flow sensor encapsulated by another polymer, polysulfone, is immersed into a hydraulic system to measure the flow rate (Figure 7a). The results suggested that the sensitivity of the proposed sensor is high enough to withstand oil pressures up to 500 bar [84]. Furthermore, PI membrane, due to its rugged nature shown in Figure 7b serves as a substitute to replace silicon oxide and nitride membranes, which leads to excellent yield. However, the PI membrane-based thermal gas flow sensors could not operate above 400 °C [82].

#### 4.1.4. Ceramics

In general, ceramics are stable and can endure high temperatures, acidic or caustic environments and are very resistant to corrosion. Therefore, ceramics are suitable for a wide range of application such as heating, melting, chemical processing, and spacecraft. Astonishingly, there are only a few ideal ceramic sensor substrates available in the field of gas flow sensing for harsh environments. Materials such as alumina and Low Temperature Co-fired Ceramics (LTCC) are nowadays increasingly used as substrates. At the very beginning, LTCC found its applications in limited areas such as military and aerospace applications. Recently, automotive applications employ LTCC based temperature sensors, because of its mechanical and chemical stability. Furthermore, LTCC has the ability to integrate heater and other structures up to 700 °C, where other materials such as glass cannot be used [85]. Although LTCC operates below the melting point, the material may soften gradually at high temperatures [86].

Consequently, another type of ceramic called High Temperature Co-fired Ceramics (HTCC) is becoming popular for various applications. HTCCs are usually fired at 1400–1600 °C, making it as a suitable ceramic candidate and a HTCC based ceramic called, zirconia could be used well in spacecraft and satellites, courtesy of its higher operating temperature and very low thermal conductivity. Despite being not abundant, processing techniques for this material were well established. A zirconia-based micro-thruster device with integrated calorimetric flow sensor was developed by Cheah et al. for use in spacecraft [87].

Furthermore, Gregory et al. designed temperature sensors based on indium-tin-oxide (ITO) ceramic, which was thermally cycled by partial oxygen exposure from 25 °C to 1250 °C and proved to be robust and versatile. Moreover, this thin-film ITO ceramic sensor can accurately measure the surface temperature of gas turbine engine. However, the material dissociates in pure nitrogen above 1100 °C, deteriorating the chemical and electrical stability [88].

On the other hand, there is a real demand for ceramic based thermal flow sensors near 1000 °C or higher. Most of the ceramic based resistive temperature sensors were developed on oxide materials such as tin oxide for gas flow detection and ${Ni}_{x}{Mn}_{y}{Fe}_{3-x-y}O_{4}$ for thermistors with temperatures less than 500 °C [89]. This entails the consideration of a wide range of ceramic metal oxides to develop temperature sensors in the future. However, characteristic problems accompanying negative temperature coefficient (NTC) such as stability and reproducibility will pose a huge task to the researchers. 

### 4.2. Insulating Substrate Materials

**Figure 8:**
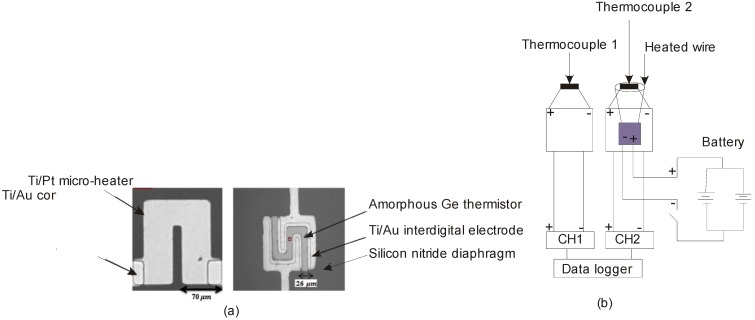
(**a**) Microscopic images of the heater and thermistor reported by Yarali et al. [90] (reprinted with permission from *Sens. Actuat. Phys. A*); (**b**) a prototype anemometer for industrial bread baking applications.

The use of insulating materials is vital in thermal flow sensor design for harsh-environment applications. The primary functions of the insulating material are the reduction of heat loss from the micro-heater to the substrate and providing an electrical insulation between the temperature sensors and the heaters [90]. Figure 8a shows an insulating membrane made of silicon nitride, over which the heater and the temperature sensors were fabricated. In most of the research works, the choice of this membrane is a silicon nitride-silicon oxide thin film [91]. These materials possess low electrical and thermal conductivity and are available in standard microelectronics fabrication. A 150-nm thick nitride membrane could bear a pressure of more than 14.5 psi. The thermal conductivities of silicon oxide and nitride reported in literature are in the ranges of 1.1–1.4 W/mK and 2.3–25 to 30 W/mK respectively [92].

Due to the excellent thermal properties of silicon nitride-based membranes, potentially dangerous exothermic reactions in micro-reactors could be studied up to 550 °C [93]. However, the membrane ruptures above this operating temperature due to the high induced thermal stress. This problem indicates that mechanical properties have to be taken into account for operation. For instance, the Young’s modulus of PECVD silicon nitride ranges from 85 to 210 GPa which is in fact lower than that of LPCVD silicon nitride, ranging from 260 to 330 GPa. An ideal insulating membrane material should have low tensile stress to avoid buckling [94]. Due to the fragile nature of silicon oxide and nitride membranes, the yield is not good and the dielectric thickness and active area are consequently limited.

Glass is an alternative substrate material to silicon nitride owing to its lower thermal conductivity and ease of fabrication [95]. In some cases, glass fibers are used as insulators and Figure 8b depicts an anemometer designed by Therdthai et al. [96] for high-pressure industrial bread-baking ovens. Glass fiber was placed between a heater and a K-type thermocouple to improve the performance and repeatability of the sensor. In fact, the sensor was able to avoid interference issue, but the sensor has a short lifespan because of damages at high temperatures. On the other hand, thermal flow sensor insulated with braided Q fiber and coated with adhesive posed no interference threat to the device and functioned longer up to 650 °C.

## 5. Recent Thermal Flow Sensors

This section provides the recent progress of the thermal flow sensors in the context of harsh environments. Table 4 and Table 5 provide the design parameters and characterization details of thermoelectric- and thermoresistive-based thermal flow sensors.

### 5.1. Thermoelectric Flow Sensors

**Figure 9:**
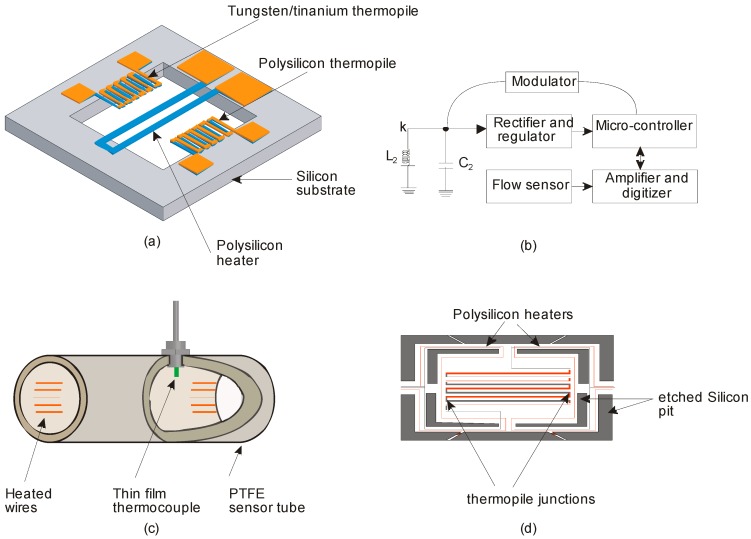
Examples of recently reported thermoelectric flow sensors, (**a**) A high-temperature thermal flow sensor with membrane, heater and thermopiles; (**b**) Hardware block diagram of the wireless flow sensor developed by Gould et al.; (**c**) Time of flight thermal flow sensor for large scale containment facility; (**d**) Top view of a thermal flow sensor showing reference and sample heaters along with five thermopile junctions on each side.

The selection of suitable sensing and other materials discussed in Section 4 leads to specific designs of flow sensors. In the last decade, a considerable amount of thermopile based flow sensors have been reported. Buchner et al. reported a high-temperature fabrication process for thermopile-based flow sensors shown in Figure 9a.

This sensor was developed for liquid level measurement, where the thermopiles are made of p-doped polysilicon and WTi, respectively. In this work, devices for different flow rates were characterized and a very short reaction time of 2.6 ms was achieved. The sensors showed a relatively high sensitivity of 9.5 m V mm^–1^ s [98]. Gould et al. proposed a polyimide based thermopile flow sensor for wireless in-situ flow measurement depicted in Figure 9b. This calorimetric sensor was tested for air flow, but is capable of operating in a wide range of media, and at high temperatures [99].

Another interesting thermoelectric flow sensor was reported by a research group at the Paul-Scherer-Institute (PSI). The sensor is suitable for the harsh environment inside the PANDA large scale containment test facility. This time-of-flight based flow sensor was made of stainless steel, Figure 9c. The sensor was designed for velocities from 0.04 to 0.3 m/s of gas mixtures of varying composition [101]. However, stainless steel sensing elements suffer from high initial and rework cost, if problem occurs. Cavicchi et al. fabricated and characterized a differential scanning calorimetric flow sensor (DSF) that consists of a suspended rectangular micro-hotplate with sample and reference zones at either end, each with a polysilicon micro heater for temperature control. A thermopile consisting of a series of successive polysilicon/metal junctions measures the temperature difference between the two zones, Figure 9d. This flow sensor was tested in various aggressive media up to 600 °C. The results suggested the possible use of pattern recognition for gas identification [102].

### 5.2. Thermoresistive Flow Sensors

**Figure 10:**
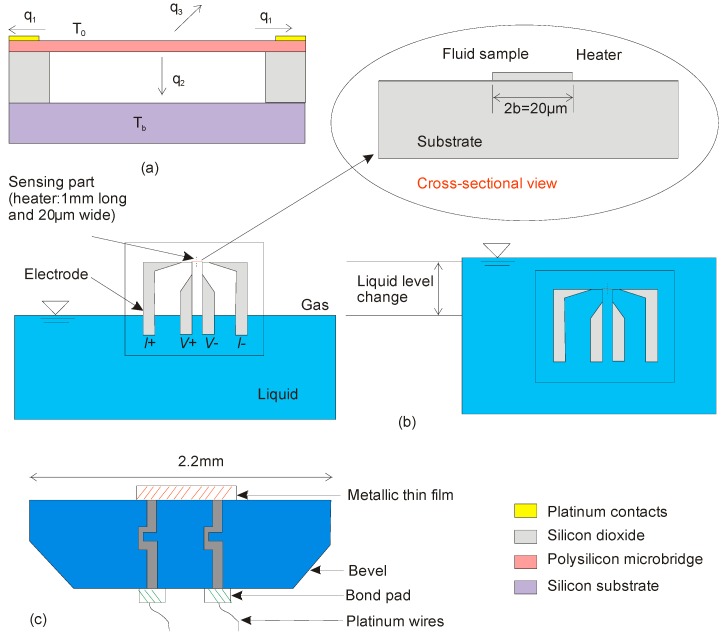
Examples of recently reported thermoresistive flow sensors, (**a**) A polysilicon micro-bridge based thermal gas flow sensor; (**b**) A volumetric thermal flow sensor for automotive fuel injection; (**c**) Schematic diagram of the operation of a point level sensor. The sensor detects the fluid phase at a specific location by measuring the temperature change of the heater where I+ and I− indicates electric current supply and V+ and V− indicates 3ω voltage output respectively.

In the recent years, a few thermoresistive flow sensors have been reported for specific applications. For instance, Mahdavifar et al. designed, fabricated and tested a polysilicon based anemometer for different gases (Figure 10a). 

The sensor showed superior sensitivity for a gas mixture of helium in nitrogen and excellent stability. In addition, it is very important to observe the dominant heat transfer mechanism at high temperature. This work has shown that heat conduction through the substrate is the key heat transfer mechanism and the maximum operating temperature of this sensor reached 526 °C.

The demand to operate the thermal sensors in aggressive fluids has been increasing. Shim and Chung proposed a micro-heater-based gas flow sensor made of silicon carbide [106]. In this work, zinc oxide, a gas sensing material has been layered over a 3C-SiC heater and characterized under nitrogen monoxide. The zinc oxide layer added with platinum has shown higher sensitivity and lower power consumption than the pure zinc oxide heater. Because, plenty of molecular oxygen is quickly adsorbed by the platinum catalyst on the gas sensing material, reducing the saturation response time.

The flow sensors reported by Mahdavifar et al. and Shim and Chung employed direct current (DC) to heat, thereby suffering from noise resistance and convection flow. Moreover, unnecessary heat loss from hot-wire to the supports results in a slow response time. Hong et al. provided a solution for this problem using an alternating current (AC) based thermal flow sensor. The authors employed the 3ω method to detect the thermal signal under the flow of R410A refrigerant and polyvinyl ether (PVE) lubrication oil (Figure 10c). The sensor was designed for high pressures of 2.35–3.82 MPa [108].

With regards to simple structure and robust performance, many industrial and automotive applications require the design of simple and compact flow sensors to be deployed inside engines. Such hot-wire flow sensor was reported by Schmid et al. (Figure 10b). Low-temperature co-fired ceramics (LTCC) has been used as the substrate material, thanks to its excellent mechanical stability. The heater element was made of Ti/Pt. The sensor was integrated into the injection nozzle in the common rail injection system and tested with diesel flow and an injection pressure of up to 135 MPa [112].

### 5.3. Thermoelectronic Flow Sensors

Thermal flow sensors operating on the principle of thermoelectronic effect have rarely been reported in the literature. However, a number of diode- and transistor-based temperature sensors for harsh conditions have been reported. The factors that determine the operation of these devices at elevated temperatures are reliability, long-term stability, sensitivity, non-linearity and repeatability.

**Figure 11:**
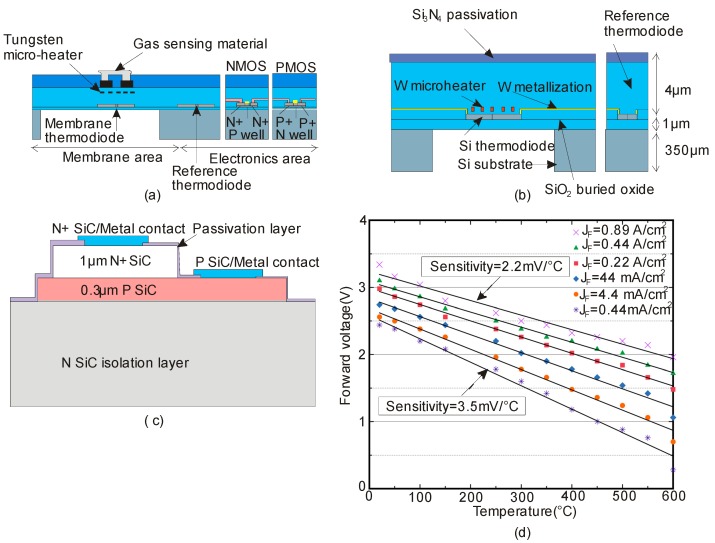
Examples of recently reported thermoelectronic sensors, (**a**) Cross-sectional view of membrane diodes, reference diode and CMOS cells; (**b**) Cross-sectional view of the micro-heater, suspended diodes and reference diode; (**c**) Cross-sectional view of 4H-SiCpn diode based temperature sensor; (**d**) Measured forward voltage versus temperature at different forward current densities of the 4H-SiC pn diode (reprinted with permission from *Appl. Phys. Lett.*).

Initially, Santra et al. reported the reliability, repeatability and long-term stability of diodes in long-term direct current operation up to 600 °C (Figure 11a). In fact, this work emphasized that piezo-resistive/piezo-junction effect should be avoided at this temperature for reliable performance [113]. De Luca et al. investigated the non-linearity and sensitivity of silicon-on-isolator (SOI) pn diodes at temperatures up to 777 °C. Figure 11b depicts the cross-section of the diode placed underneath a tungsten micro-heater, which is embedded in a thin dielectric membrane. This work has drawn three important conclusions: (i) the use of high lifetime SOI thin layer, (ii) the use of tungsten metallization for diode contacts and (iii) the use of very small junction areas [114].

Brezeanu et al. developed a SiC-based Schottky diode as a temperature sensor. For Schottky Barrier Diodes (SBD’s), the Schottky barrier and ideality factor from the forward characteristics should be linear with respect to wide range of temperature. However, the foremost concern of Schottky diode is the reliability and leakage current issues at elevated temperatures. To resolve this issue, Zhang et al. used SiC based p-n junction diode as temperature sensor (Figure 11c). In general, SiC p-n junction diode is very stable and suitable for device operation at junction temperatures beyond 800 °C. Figure 11d illustrates that the forward voltage of the device possess linear temperature dependence at all forward current densities, and it decreases with increasing temperature. In addition, at lower current density of 0.44 mA/cm^2^, the sensitivity increases to 3.5 mV/°C. Hence, a higher sensitivity can be achieved with a lower forward current level. The results reported in this work show that the IC compatible temperature sensor based on the 4H-SiC p-n junction diode is a promising technology [58].

Diodes have been used together with bi-polar junction transistors (BJTs) for temperature sensing rather than a stand-alone diode. This configuration is obtained by shorting the base and collector terminal of a transistor. This technique removes the effect of material, geometric and process variations associated with diode manufacturing process. The sensors reported in [115,116] were diode connected BJTs, and those reported in [117,118] were made of diode connected MOSFET’s with on-chip temperature measurements with improved linearity and sensitivity to thermal fluctuations. Among the works reported in this section, there is a clear evidence for the lack of studying the diode and transistor based sensors under aggressive flow media. However, with the existing transduction mechanism, materials and the sensing configuration, the thermoelectronic flow sensors for niche applications are expected in coming years.

## 6. Packaging of Thermal Flow Sensors in Harsh Environments

Current packaging technologies are one of the most limiting factors for any industrial applications that operate under harsh conditions. The selection of materials to house the sensor and their maximum surviving temperature is the first step towards packaging. Mechanical parameters such as stress, strain and other adverse influences must be sensibly controlled to ensure the proper operation of the sensor. Failure to do so will affect the long term stability and performance of the sensor. Consequently, sensor packaging is vital and the development of sensor die and packaging should be considered together for cost-effectiveness [38]. A proper mechanical, electrical or thermal interface is desirable between the sensor, inherently connected fragile components and the surroundings. Packaging of MEMS devices, particularly, thermal flow sensors is a huge task, which considers: (i) substrate material properties, (ii) metallization, (iii) die-attach and hermeticity, (iv) protective coatings and (v) signal processing and electronic circuitry.

### 6.1. Substrate Material Properties

The selection of substrate materials is significant as their properties can greatly affect the packaging performance. Coefficient of thermal expansion (TEC), thermal conductivity and thermal shock resistance are the three important thermal parameters to be taken into account for the choice of the substrates. In fact, thermal stress occurs due to the thermal expansion mismatch between various device elements and substrates and thermal cycling sometimes causes thermal shock resistance. Finally, die-attach thermal conductivity value should be maximum for safe operation levels [119].

Polyimide (PI), a commonly used substrate polymer material, suffers from long-term stability around 350 °C due to depolymerization, whereas, metals and alloy substrates suffer from oxidation around 500 °C in air. Therefore, the other options could be the use of ceramic materials as discussed in Section 4.1.4. Among ceramics, alumina offers simple fabrication and fast prototyping and is feasible for applications requiring high sensitivity. However, the lower thermal conductivity of 25 W/mK places alumina behind the other substrates. Group III nitride materials such as aluminium nitride (AlN) could be employed as substrate material. This material possesses the excellent thermal conductivity of 300 W/mK and has a close thermal expansion match with device material such as SiC leading to the reduction in thermal stress. Moreover, a high thermal shock parameter of 350 °C avoids thermal shock failures during thermal cycling.

### 6.2. Metallization

Metallization involves producing a metal thin film that functions as the interconnects of the various components on the chip. In addition, it is also used to produce metalized areas called bonding pads around the periphery of the chip. When a metallic component in a package is exposed to high temperatures, the reactivity of metal with its environment increases. Such reactivity could lead to a chemical reaction ensuing in new compound formation. Thermomechanical failure occurs due to the formation of intermetallic phases around 250 °C, reducing the mechanical strength of the interconnection system. Moreover, the electro migration induced by the flowing current is greater due to increased self-diffusion leading to open circuit failures.

In order to avoid intermetallic phase formation, conventional materials such as copper, aluminium and aluminium/nickel plated Kovar are not usually preferred for MEMS metallization processes. Instead, expensive metals are the choice. Furthermore, aluminium suffers from electromigration, which can cause considerable material transport in metals. This occurs because of the enhanced mobility of atoms caused by the direct effect of the electric field and the collision of electrons with atoms, leading to momentum transfer. Therefore, gold has been widely preferred as the interconnection metal due to its low Young’s modulus, high thermal conductivity and excellent chemical stability [120].

In some cases, multi-layer metallization stack has been tested for a stable operation. For instance, various metallization stack layers such as Al/Ni/Al, Si/Ni, NiCr, W/Cr/Ni metalized on SiC were reported by Gottfried et al. and Cole et al. [121,122]. Despite possessing low specific contact resistance and excellent integrity, the nickel silicide layer formed by annealing at 950 °C is susceptible to air and suffers oxidation problem. To address this issue, Baeri et al. reported a metallization scheme that adds a conduction metal layer (Au/WTi) on the top of nickel silicide layer. The complete metallization stack was tested in vacuum and in oxygen environment and demonstrated that the multi-layer scheme performed well around 950 °C for 100 h.

### 6.3. Die-Attach and Hermeticity

Die attach involves attaching or bonding a die or chip to a substrate, package or another die. This process is application specific and can be implemented in different ways. In general, an ideal die-attach material should possess: (i) zero debonding or delamination i.e., the die and the substrate should be adhered together, (ii) self-resilience to deliver good stress reduction behaviour so that induced internal stresses are reduced, (iii) high thermal conductivity to maintain safety operating levels and reduce heat dissipation, (iv) excellent resistance to corrosion and (v) better reworkability.

Metal alloys, organic or inorganic adhesives are usually employed as intermediate bonding layers in die-attach processes. Metal alloys encompass all forms of solder, including eutectic and non-eutectic. Organic adhesives consist of epoxies, polyimide and silicones. The choice of a solder alloy is governed by its melting temperature and mechanical properties. Compared with organic adhesives, solder alloy provides strong attachment of die to package with less stress [123]. In addition, die-attach materials have to be cured or processed at a temperature that does not damage the die and establish reliability over a long period of time. As the die-attach is in close contact with the substrate, the materials should be mechanically, thermally and electrically stable at high temperatures. Moreover, another aspect would the stability of interface between die and substrate or between metallization of die and substrate. These aspects should be taken into consideration for die-attaching materials and proper consideration leads to achieving an excellent packaging technique. Hermeticity is key for physical protection and in some cases, it determines the device performance. Organic materials are not good candidates for hermetic packages. For almost all high-reliability applications, the hermetic seal is made with glass or metal similar to the die-attach materials. Finally, the packaged sensor should be miniaturized and light. Cost effectiveness should also be considered for commercial market.

### 6.4. Protective Coatings

It is common that thermal flow sensors are exposed to potentially corrosive environments. Therefore, another aspect that must be considered in protecting them is providing coatings. The coatings included are standard silicon based passivation layers. This protective coating should be able to cover lead frame, bond wires, bond pads, chip surface and edges from the media. Thus, the lifetime and performance of the flow sensors are known by investigating the corrosion rate of these layers in acidic, alkaline and in neutral fluid medium. Eriksen and Dyrbye et al. reported that silicon carbide when exposed to alkaline media has shown low corrosion rate $\left(2.6\;Åh^{-1}\right)$ than silicon nitride $\left(70\;Åh^{-1}\right)$ and silicon oxide $\left(1000\;Åh^{-1}\right)$ at 140 °C [124]. Moreover silicon carbide has been used as a coating material for MEMS strain sensors [15], atomizers [125], MEMS actuators [126] and capacitive flow sensors [127]. A comprehensive protection coatings for thermal flow sensors have rarely been reported and this motivates the researchers around the globe to fill this niche gap in the coming years.

### 6.5. Signal Processing and Electronic Circuitry

Thermal flow sensors operating in hostile conditions are needed, to avoid disasters caused by structural and system failures at elevated temperatures. However, most of flow sensors on the market do not meet this need, because the wires or cables for signal conditioning require either physical contact or a battery for power supply. None of these components can withstand the high temperature. Moreover, batteries limit the sensor lifetime and the operating temperature range. For instance, in aircraft applications, a conventional soft wire/cable will be dismantled by a temperature of 450 °C in less than one hour. One possible alternative to the conventional soft cables is a hard alumel cable, which does not even degrade after 10,000 h, thereby improving the lifetime [128]. Another possible solution is the use of wireless operating modes. A virtual battery powered by electromagnetic radiation could replace the conventional electrochemical batteries as power sources. In the last decade, a vast amount of works have been done on RF powered high temperature pressure sensing [129,130,131], chemical sensing [132] and humidity sensing [133].

The capability to process, amplify and even wirelessly communicate signals directly from the point of sensing would benefit many applications. Therefore, eliminating wires/cables associated with high-temperature flow sensors will reduce weight and increase the reliability of the sensor. In addition, such electronics needs to be small in size, of low weight and non-contact to operate for a longer time. SiC based electronic circuits such as diodes, Junction Field Effect Transistors (JFETs) and Metal-Oxide Semiconductor Field Effect Transistors (MOSFETs) have successfully demonstrated long time operation at high temperatures [134]. However, further developments are necessary for the communication between the electronic circuitry and the sensing module.

### 6.6. Recent Packaging Strategies of Thermal Flow Sensors

Packaging of thermal flow sensors has to meet two requirements: (i) the sensor should be in contact with the ambient air and (ii) good isolation between the sensor, processing circuit and surroundings to avoid unwanted external influences. Moreover, the packaging technique should offer high reliability and stability at low cost [135].

**Figure 12:**
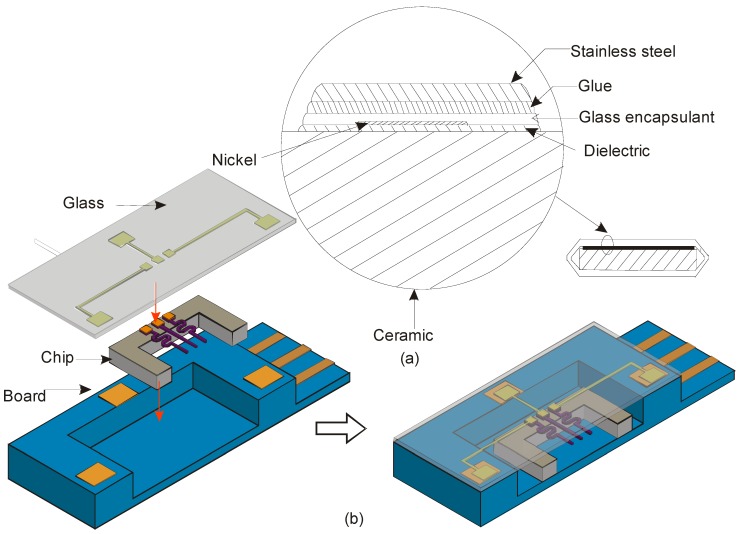
Example of packaging a thermal flow sensor, (**a**) A thermal flow direction element in thick film technology packaged for aggressive media and (**b**) Schematic cross-section of entire assembling of wire sensor developed using SOI technology for harsh aggressive media.

In the late 1990s, mounting technology with small dimensions were not available for commercial silicon based sensor. Thick film technology was more common for thermal sensors along with packaging. For instance, Dyrbye et al. proposed a packaging technique, where the ceramic substrate holds a thin insulating membrane. The nickel sensor encapsulated by a glass layer of 6 μm (Figure 12a). Stainless steel of 100 μm thickness is glued to the sensing element for protection and provides a stable and short thermal path to the aggressive media. The packaged sensor was able to detect a frequency of 0.05–33 Hz when tested with water flow [136]. However, this packaging suggested the inadequacy of chemical stability and the lack of maturity of the packaging schemes at that time. This outcome led to the development of various packaging approaches, some of those are discussed below. 

Wire bonding is one of the early adopted packaging methods, which suffers from fatigue and creep deformation at high temperatures. Due to the deformation, the wire bonds break leading to failure of the device. Therefore, wire bonding has been replaced by other packaging techniques to meet the primary requirements of application specific designs.

Flip-chip packaging involves interconnecting the sensors to external circuit (e.g., a circuit board or another chip or wafer), with solder-bumps that are deposited onto chip pads. Sosna et al. developed a flow sensor based on a thermoelectric principle [52] and packaged it using flip-chip technology. Reflow-soldering process was employed to connect the sensor electrically and mechanically to a PCB. Silk screen printing was used to deposit tin-solder (96.5% tin, 3% Ag, and 0.5% Cu). In fact, both silicon wafer alignment technology and flip-chip technology enables the precise alignment of tin-solder on the silicon wafer. Tin is deposited by laminating, exposing and structuring a dry-resist on the silicon wafer, followed by reflow soldering to form solder-bumps and removing the dry-resist wet-chemically. Finally, the sensors were flip-chip bonded on a PCB. The packaged thermoelectric sensor was tested under air flow in the range of 0.4–12 standard litres per minute (SLPM). Its high process temperature of 1050 °C suggests suitability for industrial and automotive applications. However, flip-chip technology is not appropriate for thermal sensors that are designed to be in open contact with the environment. Only capped thermal sensors can take the full advantage of implementing this technique [123].

In the last decade, research on packaging, specifically for thermal flow sensors are few. Measuring high flow rates with a bypass was a common solution reported in the literature. However, Billat et al. developed a simple and robust thermal flow sensor, which can detect high flow rates up to 500 L/h in water (Figure 12b). The encapsulation technique was implemented in such a way that the sensor is directly placed in the fluid channel wall and tested with aggressive media.

In this packaging strategy, the electric connection paths between the silicon chip and the processing circuit is made of ceramic or glass die acting as the first housing. Mechanical isolation and thermal interface were obtained by connecting the chip to a ceramic die with a defined gap of 10 μm. The reproducibility obtained with this packaging is good enough in harsh environments, but the packaging itself should be tested with the flow of other aggressive fluids such as methane or propane before deploying it in any industries [68]. 

Recently, Brushi et al. proposed three techniques to package a thermal gas flow sensor to study the sensitivity. They are: (i) chip-inside-channel (CIC), (ii) half pipe (HP) and (iii) local conveyor (LC). These three packaging methods offer low-cost and all are compatible with chips of standard shape and dimension. 

**Figure 13:**
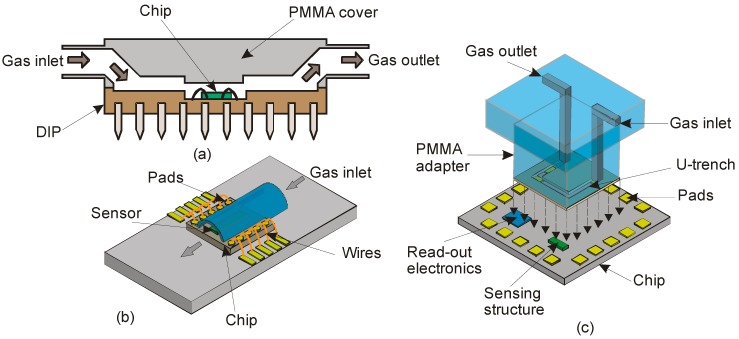
Schematic cross-sectional view of the experimented packaging strategies for a thermal gas flow sensor, (**a**) Whole chip inside the flow channel (CIC), (**b**) Half-pipe (HP) and (**c**) PMMA local conveyor (LC).

Figure 13a shows a CIC technique where a PMMA cover containing two channels for inlet and outlet gas flow is glued to the top of the DIP case by epoxy resin. The chip and all the bonding wires are placed inside the channel for packaging. However, with an aggressive gas flow, the bonding wires may be damaged. In the HP technique, a cylindrical metal pipe of 2.4-mm diameter is milled and positioned on the chip with the bonding pads on each sidewalls of the chip. A channel area of 0.8 mm^2^ covers the sensing structure of the chip. Gas flow is only allowed into this area, as depicted in Figure 13b. Despite the reduction in channel size covering the sensing area alone, gas leakage occurs due to the chip border, where maintaining the bond pads became difficult. Finally, in the LC technique, a U-shaped trench is formed by milling and a PMMA adapter is pressed against the trench that rests on the chip surface as shown in Figure 13c. In addition, the sensing structure and read-out electronics were placed inside the trench and the bonding pads outside to avoid the undesirable influences during gas flow. This configuration is advantageous over the other two, as the area of the channel including the sensing structures can be varied by simply changing the width or depth of the trench [137]. Although these packaging configurations give better sensitivity with gas flow measurements, the operation temperature is only below 160 °C.

## 7. Conclusions and Future Challenges

Various studies on the development and characterization of thermal flow sensors in different materials have been carried out in the last decade. The reported high operating temperature and working pressure of the flow sensors demonstrate their potential use in harsh environments. Despite many challenges involved in the development of thermal flow sensors and their operating conditions, they continue to proliferate and the market appears to be promising because of the following reasons: (i) a well-established understanding of sensing mechanisms of thermal flow sensors, (ii) new and better-quality materials are coming to market, suggesting the improvement in device fabrication techniques, (iii) application-specific thermal flow sensors are becoming more common than the development of sensors for generic uses. However, the design and development of thermal flow sensors for petrochemical and aerospace applications seems more challenging than those for domestic and biological applications. There are many technical challenges which make the design and operation more cumbersome, which include:

Firstly, selecting materials with the correct heat transfer, strength, and electrical properties is important to achieving an ideal sensor system design. Each element of the system must perform independently and in combination with another to optimize the sensor performance and lifespan. Thermal diffusivity, thermal resistance and shock, fatigue resistance over a wide range of temperatures and cost are some of the essential properties to be taken into account. These properties can all vary significantly with environmental extremes, so care must be taken to analyze the material properties versus the target operating conditions. 

In addition, it is crucial to select a material that can reduce the cost of all components and easily integrate sensor elements on the substrate. In this aspect, platinum may not be the best choice compared to Si, SiC and SiO_2_. In most cases, SiC is a good choice as it can be deposited on the Si substrate and SiC is superior in performance compared to metallic ones. Furthermore, the operating temperature of SiC lies in the vicinity of >1000 °C. However, SiC suffers from oxidation problem that lead to long-term drift. Moreover, the influence of high temperature on semiconductors could drastically affect the electrical behavior, i.e., thermal stability at high temperatures is an important criterion and should be well considered for the material choice. Hence, semiconductor materials with large bandgap and lower intrinsic carrier density at high temperatures should be considered. In doing so, 4H-SiC and 6H-SiC polytypes retain better bandgap than their counterparts, Si and GaAs, respectively. However, for 4H-SiC and 6H-SiC to gain much prominence, low defect wafers should be produced in the market.

Secondly, degradation of contact electrodes is a major concern. Contact electrode materials such as tungsten, molybdenum, nickel and titanium suffer degradation problem at extreme temperatures, leading to device failure. The possible option could be the use of electrodeless devices, but stability is not possible. Therefore, semiconducting metal oxides such as SiO_2_: Sb or conductive ceramic oxides could be the best candidates with respect to these two material miscellaneous challenges.

Thirdly, exposure of thermal flow sensors and its components to harsh conditions can lead to the failure of the device. Therefore, robust packaging methods must be further established for the integration, protection, reduction of electrical and mechanical noise and sensor lifespan. More precisely, encapsulation, bonding, electrical contacts and interconnects should be considered. In addition, development of metallization and di-electric materials in compatible with SiC, or SiO_2_: Sb should be developed to prevent interdiffusion at the boundaries or alloying of materials.

Lastly, the development of thermoelectronic flow sensors in the last decade has not penetrated much into the MEMS market as discussed in Section 5.3. However, researchers around the globe have shown glimpses of interest in the design of temperature sensors based on diodes and transistors. The group III nitrides such as GaN and AlN with their popular uses in optoelectronic devices could join cubic silicon carbide as a device material for favoring thermal flow sensors in the upcoming years.

## Figures and Tables

**Table 1:** Summary on the impact of high temperature on various thermal sensors.

Temperature Sensor	Effect of High Temperature	Maximum Working Temperature
Thermistor [26]	Number of charge carriers and conductivity increases	1050 °C
Thermopiles [50]	Magnitude of Seebeck voltage increases	~1000 °C
pn junction diode [58]	Forward voltage drop decreases and leakage current increases exponentially	Beyond 600 °C
Schottky diode [59]	Forward voltage drop decreases and reverse current increases with $T^{2}$	700 °C
BJT [60]	Base-emitter voltage decreases at collector current and current amplification increases with $T^{x}$ (1 < x < 2)	400 °C
JFET [61]	Channel mobility decreases with $T^{3/2}$ and pinch-off voltage increases	500 °C
MOSFET [60]	Channel mobility decreases with $T^{3/2}$, leakage current of pn junctions increases exponentially and threshold voltage decreases	650 °C

**Table 2:** Key advantages and drawbacks of commonly employed heater metal and alloys at high temperatures.

Material	Key Advantages	Limitations
Tungsten	Mechanically strong and high TCR (0.004/°C)	Poor resistance to oxidation at high temperatures to many gases
Platinum	Good oxidation resistance and good TCR (0.003/°C)	Mechanically weak at high temperatures
Nichrome	Less expensive and high temperature corrosion resistant	Not self-supporting
Platinum-iridium alloy	Good oxidation resistance and high tensile strength than platinum	Low TCR (0.00085/°C)
Platinum-rhodium alloy	Higher TCR than platinum-iridium	Not as strong mechanically as platinum-iridium

**Table 3:** Material properties of various semiconductors for high temperature applications (adapted and updated from [26,60,81]).

Properties	Si	3C-SiC	4H-SiC	6H-SiC	GaAs	GaN	AlN
Band gap (eV)	1.12	2.36	3.23	3.05	1.4	3.4	6.2
Thermal Expansion coefficient $\left(10^{-6}K^{-1}\right)$	2.6	2.9	-	4.2	5.7	5.6	4.5
Lattice constant (nm)	0.543	0.435	0.307	0.308	$0.3189\;a_{0}$	$0.5185\;c_{0}$	$0.311\;a_{0}$
Thermal conductivity ${(\mathrm{Wcm}}^{-1}K^{-1})$	1.5	3.3-4.9	3.7	4.9	0.46	1.3	3
Density $\left({\mathrm{gcm}}^{-3}\right)$	2.33	3.21	3.21	3.21	5.32	6.15	3.25
Electronic maximum operating temperature (°C)	150	600	750	700	350	>700	>700
Relative dielectric constant	11.8	9.72	9.7	9.66	12.5	11	10
Young Modulus (GPa)	130–185	310–550	390–690	390–690	85.5	271	302–348
Physical stability	Good	Excellent	-	-	Fair	Good	Good
Hole mobility $\left({\mathrm{cm}}^{2}V^{-1}s^{-1}@N_{A}=10^{16}\;{\mathrm{cm}}^{-3}\right)$	480	40	115	90	400	250	14
Electron mobility $\left({\mathrm{cm}}^{2}V^{-1}s^{-1}@N_{D}=10^{16}\;{\mathrm{cm}}^{-3}\right)$	1430	800	(⊥c-axis:400)	(⊥c-axis:400)	8500	1250	-
Breakdown field ${(10}^{5}{\mathrm{Vcm}}^{-1}@N_{A}=10^{17}\;{\mathrm{cm}}^{-3})$	3	>15	(∥c-axis:3.0)	(∥c-axis:3.2) (⊥c-axis>1)	6	>50	>50
Saturation electron velocity ${(10}^{7}{\;\mathrm{cms}}^{-1})$	1	2.5	2	2	1	2.2	1.4

**Table 4:** Typical research results of thermoelectric flow sensors.

Configuration	Materials	Fluid	Flow Range	Sensitivity	Power Consumption	Maximum Temperature	Application
Time of flight [50]	Stainless steel	Water	0.5–5 gal/min	-	-	>300–1000 °C	Nuclear plant
Calorimetric [52]	Si, Poly-Si and WTi	Air	0.4–12 Slpm	-	-	~1050 °C	Industrial
Calorimetric [83]	Poly-Si, WTi and quartz	Water	0−20 Q/μL/S	2.77 mV/K	-	-	Hydraulic
Hot-wire [96]	Ni-Cr and glass fiber	Air	0.1–4 m/s	Depends on surface contact between heater and thermocouple	-	650 °C	Bread-baking oven
Hot-wire [97]	Si and WTi	Air	0–0.7 Slpm	−1.3 mV/℃	17.99 mW	300 °C	Emission control
Calorimetric [98]	Poly-Si and WTi	Water, isopropanol	0–15 Q/mg/S	9.5 mv/mm	-	~800 °C	High pressure liquid flow/harsh condition
Calorimetric [99]	SiN and PI	Air	0–50 m/s	1.8 mV/K	1.5 mW	350 °C	Wireless insitu flow
Calorimetric [100]	Poly-Si/Al	Nitrogen	0–8 m/s	-	30 mW	~500 °C	Gas sensing
Time of flight [101]	Stainless steel	Mixture of helium, air and steam	0.04–0.3 m/s	-	-	-	Nuclear plant
Calorimetric [102]	Poly-Si/Pd	Methanol	0−100 μmole/mole	3 μmole/mole	-	600 °C	Pattern recognition
Pseudo-calorimetric [103]	Poly-Si/Al	Nitrogen	0–200 Sccm	-	-	-	Gas sensing/High pressure

Slpm—standard litres per minute; sccm—standard cubic centimetres per minute.

**Table 5:** Typical research results of thermoresistive flow sensors.

Configuration	Materials	Fluid	Flow Range	Sensitivity (S)	Power Consumption	Maximum Temperature	Application
Calorimetric [45]	Pt	Nitrogen	0–4 m/s	-	2–20 mW	500 °C	Explosive gas sensing
Calorimetric [46]	Yttria Zirconia	Nitrogen	0–40 sccm	0.15 mΩ/sccm	2 W	>1000 °C	Spacecraft
Calorimetric and anemometric [68]	Glass/ceramics and Si	Air, water	Air: 0–110 m/s Water:500 1/h	0.075 V/m/s for a velocity of 40 m/s	250 mW in	-	High-flowrate
Calorimetric [72]	Poly-Si	Propane in air	0.01–0.8 Vol %	-	15–20 mW	800 °C	Catalytic Micro reactors
Hot-film [84]	PI, polysulfide and Au	Oil	25–75 L/min	High dynamical sensitivity	Depends on flow rate	-	High pressure hydraulic systems
Calorimetric [104]	SiC and porous Si	Air	0–4 m/s	$S=\sqrt{V}$	60 mW	Melting point of SiC	-
Hot-wire [105]	Si and Poly-Si	Helium fractions in Nitrogen	Up to 700 ppm	0.34 mΩ/ppm	4.3 mW	526 °C	Safety sensing
Hot-wire [106]	SiC, Si, ZnO/Pt	Nitrogen monoxide	0.046–0.223 ppm	~1.7 @ 500 °C	10.3 mW	500 °C	Aircraft
Hot-wire [107]	LTCC	Diesel	0–117.285 m/s	-	1.8 W	-	Automotive/High pressure
Hot-wire [108]	Glass, Au/Cr	R410A refrigerant and ethanol	35 mL of liquid added to 50 mL beaker	1.4 at 5000 Hz for ethanol flow	-	80 °C	Oil industry/High pressure
Hot-wire [109]	Stainless steel	Ethanol	3.4–3.8 cm/s	-	-	250 °C	Liquid level
Calorimetric [110]	Graphite, Au	Hydrogen and methane	$\begin{array}{l} H_{2}:4\\ {\mathrm{CH}}_{4}:8\mathrm{slpm} \end{array}$	-	50–100 mW	255 °C	Fuel cell
Calorimetric [111]	Si, Pt	Methane	-	-	0.3–1.6 W	700 °C	Micro reactors
